# Acute psychosis unveiling diagnosis of hypothyroidism: A case report

**DOI:** 10.1016/j.amsu.2022.104565

**Published:** 2022-09-07

**Authors:** Himal Bikram Bhattarai, Gehendra Jung Kunwar, Ashes Rijal, Sangam Shah, Manish Uprety, Ayusha Subedi, Prabesh Bikram Singh, Santosh Khanal, Bidhan Bikram Shah, Ashim Rupakheti

**Affiliations:** a Gandaki Medical College, Teaching Hospital and Research Center, Pokhara, Nepal; b Tribhuvan University, Institute of Medicine, Maharajgunj, Nepal; c Kathmandu University School of Medical Science, Dhulikhel, Nepal; d Manmohan Memorial and Community Hospital, Jhapa, Nepal; e Kathmandu Medical College Teaching Hospital and Research Center, Kathmandu, Nepal

**Keywords:** Psychosis, Hypothyroidism, Diagnosis

## Abstract

**Introduction:**

Hypothyroidism is a common condition in the general population that presents a wide array of medical, neurological and psychiatric symptoms. However, hypothyroidism rarely leads to acute psychosis, termed myxedema psychosis (MP) and is often missed by many physicians.

**Case presentation:**

Here we report a case of a 36-years-old female who presented with a one-week history of abnormal behavior, delusions and hallucinations. Investigations revealed a high thyroid-stimulating-hormone (TSH)of 78.60 mlU/mL and low free thyroxine (FT4) of 0.64 pmol/L. Diagnosed with hypothyroidism, she was treated with oral thyroid hormone replacement (l-thyroxine 75 μg/day) with antipsychotics and her symptoms settled within days. She was discharged off antipsychotics and advised to adhere to thyroxine replacement and to follow up for Thyroid function test (TFT).

**Discussion:**

Myxedema psychosis is an uncommon manifestation of the common endocrine disease hypothyroidism. The atypical nature of presentations occasionally complicates diagnostics. When approaching a 'first-episode psychosis,’ it is essential to perform a complete organic screen consistently.

**Conclusion:**

Acute myxedema madness should be considered in the differential diagnosis of acute psychosis in patients with hypothyroidism.

## Highlights

• Hypothyroidism rarely leads to acute psychosis, termed myxedema psychosis.
• Acute myxedema madness should be considered in the differential diagnosis of acute psychosis in patients with hypothyroidism.
• When approaching a 'first-episode psychosis,’ it is essential to perform a complete organic screen consistently.

## 1 Background

Hypothyroidism, the common clinical condition of thyroid hormone deficiency, is the second most common endocrine disorder among women [1]. The most common sign and symptoms of hypothyroidism are bradycardia, hair loss, muscle cramps, voice changes, dry skin, non-pitting edema, constipation, cold sensitivity, and fatigue. Moreover, changes in mental status can also be associated with hypothyroidism ranging from mild cognitive impairment to full-fledged myxedema coma. Uncommonly, changes in mental status can involve delusions and/or hallucinations and have been described in the literature as myxedema madness [2]. However, many physicians often overlook hypothyroidism as a cause to the acute onset of psychosis. Here we present a case of hypothyroidism that came to clinical attention due to acute psychotic symptoms consisting of delusions and hallucinations (auditory and visual both). This case report has been reported in line with the SCARE Criteria [3].

## 2 Case presentation

We present the case of a thirty-six-year-old married lady who was admitted to our hospital with a one-week history of abnormal behavior. Her family member stated that she had labile mood, aggression, combativeness, delusions and hallucination. They reported her seeing the rope in the house as snake. They also reported delusions that she had of her family members trying to kill her after she heard unusual voice in her head. Prior to the current presentation, she was in her usual state of health. There was no prior history of depression, mania or psychosis and no family history of psychiatric illness. She had neither a lifetime history of substance use nor significant past surgical and medical history.

At the time of presentation, her vital signs were a blood pressure of 90/60 mmHg, temperature of 36.8 °C, and a pulse of 50 beats per minute. She was oriented to time, place, and person but avoided eye contact. She looked anxious with irritable affect. Her speech was coherent and relevant, but of low tone, volume, and rate. She had poor insight and paranoid thoughts. There were no findings of dry skin, voice hoarseness, nonpitting peripheral edema, or other apparent signs of hypothyroidism. Neurological examination was unremarkable, as with other systemic exams. Her ECG revealed Sinus Bradycardia with Heart rate of 54bpm/regular. Echocardiography showed Normal sized cardiac chamber with normal valvular anatomy. Her Renal function test including the electrolytes were within the normal limit. CT scan of head was unremarkable. However, Laboratory investigations revealed a high thyroid-stimulating hormone (TSH) of 78.60 mIU/mL (0.39–6.16 mIU/mL) and low free thyroxine (FT4) of 0.64 pmol/L (0.8–2 pmol/L). Her thyroglobulin antibodies were negative. Her LFT revealed AST value of 111.3U/l (5–42U/l) and ALT value of 85.55 U/l (5–40 U/l). She was than admitted to Medicine ward where oral therapy with thyroid hormone replacement (l-thyroxine 75 μg/day) was initiated. Similarly, our colleagues from psychiatry suggested adding Olanzapine only as needed for agitation. Following Thyroid replacement therapy her condition gradually improved. After Day 2 of admission she report of not hearing any voice in her head but still she was irritable. After 4th day of admission she lack any delusion and hallucination. Her mood was also significantly improved lacking any irritable or labile mood. When asked regarding the symptoms of delusion and hallucination, she state she doesn't remember of the episode in past. Hence after a week of admission she was almost at baseline and was discharged home off antipsychotics. Upon discharge, she was advised to follow the thyroid function test and adhere to thyroxine replacement. After a month of discharge when she came for a follow-up. Her TFT were within the normal range and she demonstrated normal mood, insight, and mental status.

## 3 Discussion

This hypothyroidism facade (Myxedema Psychosis) frequently masquerades as the primary psychotic disorder, especially in patients with no prior history of hypothyroidism [4,5]. Besides that, other characterizing symptoms of hypothyroidism may be completely missing at the time of presentation, as in our case, and the patient presents with nothing but psychiatric complaints [[6], [7], [8]] [[6], [7], [8]] [[6], [7], [8]]. As a result, it is crucial to highlight that many people with psychiatric problems may also have endocrine dysfunction, and that the absence of typical hypothyroidism signs and symptoms does not rule out the diagnosis [9,10]. The patient's drug use could be the cause, but since there were no drugs in the urine, this was ruled out. Alcohol intoxication could also be the cause, but since he occasionally drank alcohol and did not exhibit any tremors, seizures, or cravings, this was also ruled out.

The term “Myxedema” was first used to address the non-pitting edema observed in some patients with hypothyroidism by William Ord. The Committee on Myxedema of the Clinical Society of London first established the relationship between hypothyroidism and psychosis in 1888 [11]. It found the delusions and hallucinations in nearly half of the cases out of 109 patients presenting myxedema. However, the term “myxedema madness” was first introduced to the literature in 1949 by Asher describing 14 patients, all having myxedema and psychotic changes. Among them, nine patients had a complete recovery, two patients had partial improvement, one showed no change, and two died with thyroid treatment [12].

Thyroid replacement therapy is the mainstay of treatment for myxedema psychosis patients. Some of them can return to their original state of good mental health with this therapy, but this is not always the case [12]. Short-term antipsychotic treatment, typically with risperidone and haloperidol, is critical for assisting treatment and promoting faster recovery [2,4,6,9]. Fortunately, our patient also reverted to her original health state with the thyroid hormone replacement therapy and the anti-psychotic medications. The latter was stopped while discharging her from the hospital and we asked her to follow up after a week.

## 4 Conclusion

The most prevalent hormonal condition is hypothyroidism, although it is far from simple to obtain an accurate diagnosis in a patient. We propose that acute myxedema madness should be considered in the differential diagnosis of acute psychosis in patients with hypothyroidism. Numerous symptoms and signs are known to manifest that are associated with hypothyroidism. This is a case report and thus the findings are not generalizable to a larger population.

## Provenance and peer review

Not commissioned, externally peer-reviewed.

## Ethical approval

None.

## Please state any sources of funding for your research

No funding was received for the study.

## Author contribution

GJK and HBB wrote the original manuscript, reviewed, and edited the manuscript. HBB, GJK, AR, SS, MU, AS, PBS, SK, BBS, AR reviewed and edited the manuscript.

## Registration of research studies

1. Name of the registry: None
2. Unique Identifying number or registration ID: None
3. Hyperlink to your specific registration (must be publicly accessible and will be checked):

## Guarantor

Himal Bikram Bhattarai.

## Consent

Written informed consent was obtained from the patient for publication of this case report and accompanying images. A copy of the written consent is available for review by the Editor-in-Chief of this journal on request.

## Declaration of competing interest

Authors have no conflict of interest to declare.
